# TR Self-Adaptive Cancellation Based Pipeline Leakage Localization Method Using Piezoceramic Transducers

**DOI:** 10.3390/s22020696

**Published:** 2022-01-17

**Authors:** Yanbin Mo, Lvqing Bi

**Affiliations:** 1 School of Physics and Telecommunications Engineering, Guangxi Colleges and Universities Key Laboratory of Complex System Optimization and Big Data Processing, Yulin Research Institute of Big Data, Yulin Normal University, Yulin 537000, China; moyanbin9559@163.com; 2 Department of Electronic Engineering, Xiamen University, Xiamen 361005, China

**Keywords:** time reversal, localization, PZT transducer, negative pressure wave, pipeline leakage

## Abstract

In this paper, we propose a novel time reversal-based localization method for pipeline leakage. In the proposed method, a so-called TR self-adaptive cancellation is developed to improve the leak localization resolution. First of all, the proposed approach time reverses and back-propagates the captured signals. Secondly, the time reversed signals with the various coefficients are superposed. Due to the synchronous temporal and spatial focusing characteristic of time reversal, those time reversed signals will cancel each other out. Finally, the leakage location is distinguished by observing the energy distribution of the superposed signal. In this investigation, the proposed method was employed to monitor a 58 m PVC pipeline. Three manually controllable valves were utilized to simulate the leakages. Six piezoceramic sensors equipped on the pipeline, recorded the NWP signals generated by the three valves. The experimental results show that the leak positions can accurately revealed by using the proposed approach. Furthermore, the resolution of the proposed approach can be ten times that of the conventional TR localization method.

## 1. Introduction

Structural health monitoring (SHM) is a newly developed research field devoted to the monitoring and assessment of structural health and durability. SHM is especially useful for remotely monitoring the health of high-profile mechanical systems such as spacecraft, ships, offshore structures, and pipelines where onsite monitoring is difficult or even impossible [1]. Optic fiber bragg grating is employed for the applications for SHM [2,3,4], such as monitoring the load level of the bolts [5,6] and the detection of hoop-strain in the pipeline [7,8,9]. Another SHM systems are based on lead zirconate titanate (PZT) transducer. For the PZT based passive sensing, the PZT transducers are employed to acquire the stress signals from impact or leakage [10,11,12,13]. Furthermore, the active sensing based on PZT is applied more widely [14,15]. For example, the monitoring of debonding in the structures [16,17,18], the pipeline damage detection [19], the grouting compactness monitoring of post-tensioning tendon duct [20,21], the pre-stress monitoring of rock bolts [22], the evaluation of the fatigue damage in the modular bridge expansion joints [23], structure damage imaging [24,25], and real-time monitoring of soil compaction [26].

As an important investigation field of SHM, pipeline leakage monitoring is attracting extensive attention [27,28] since the pipeline leakage caused numbers of catastrophic accidents every year around the world. The detection methods of the pipeline leakage have been explored in numerous studies in the available literature. The representative pipeline leak localization methods include the transient model method [29,30,31], the magnetic flux detection method [32,33], the acoustics method [34,35,36,37], the image analysis method [38], the hybrid method [39,40], the negative pressure wave (NPW) method [41,42], etc. For example, Ni et al. [31] compared the leak localization based on the particle swarm optimization theory with that based on the transient model. Yan et al. presented the magnetic flux leakage detection technique which uses magnetic sensitive sensors to detect the magnetic leakage field of defects on both the internal and external surfaces of pipelines [32]. Bian et al. developed a leakage localization method which analyzes the space-time correlation of the ultrasonic signals of the leakage hole [37]. Su et al., detected leaking defects by using an image segmentation method [38]. Zhang et al. [40] proposed a hybrid leak detection technology which combined the negative-pressure wave method and the transient modeling method. Liu et al. [41] developed a novel leakage detection approach based on the amplitude attenuation model of dynamic pressure waves. Although the aforementioned technologies are capable of detecting the existence of leakages, they may suffer from high computational cost [31], expensive equipment [32,33], or large location error [41]. For example, the location error of the hybrid leak detection technology is about 10%. Although the transient model method has a high localization accuracy, its complex models mean a lot of training and expensive cost which prevent its wide applications. Compared with other methods, the NPW method is widely utilized due to its simple operation and high accuracy.

When a leak occurs, a negative pressure wave propagates toward the two ends of a pipeline from the leak position. After recording the NPW signals via the sensors mounted on the outside layer of the pipeline, the signal processing algorithms are utilized to analyze the NPW signals, in order to localize the leakage points. For instance, Jia et al. [42] developed a leakage localization approach which detected the NPW by measuring the hoop strain. Zhu et al. [43] localized the leakages by calculating the arriving time of the NPW and analyzed the localization accuracy. Hou et al. [8] developed a compound Simpson formula and dichotomy searching-based leak localization formula. Zhou et al. [44] developed an improved spline-local mean decomposition to process the negative pressure wave signal, and revealed the leak by using the convolutional neural network to analyze the processed signal. Li et al., developed a novel localization algorithm based on the attenuation of negative pressure wave [45]. Those methods aimed to improve the location error via suppressing the noise [44,45]. No matter that the localization methods are based on the time delay estimation [42,43,44] or the attenuation of negative pressure wave [45], most of them merely revealed the leakage positions. Other acoustic signatures such as −3 dB width which sets a boundary limit between leakage points and non-leakage points, cannot be observed from the results obtained by using the existing methods. However, Ing et al. proved that −3 dB width and the maximum peak which are the two main acoustic characteristics can be identified by using the time reversal (TR) localization method at the same time [46].

In the computational time-reversal localization [47], the signals are computationally re-radiated into the area of interest [48,49,50] in a computer [48]. In the computational process, the TR methods generate grids in the monitoring area, and obtain the signals at the grids by convoluting the time reversed signal with channel impulse response in time domain. Due to the spatial reciprocity, TR techniques can force the signals to focus at their sources. The sources’ locations can be revealed by plotting the distribution of the TR signals’ energy [51,52]. Recently, time reversal technology is studied for non-destructive testing and SHM [53,54]. For example, Amitt et al. performed a computational TR run, and obtained an identification solution at the reference time [55]. In concrete model, the peak of the TR signal is utilized to identify the source’s position [56,57]. Zhao et al., built the defect image map by using the maximum of the time reversal field [58].

However, for the passive detection, such as the pipeline leakage detection, the time reversal localization suffers several issues. Due to the long signal duration which is the result of the superposition of incident and reflected NPW, although the conventional TR localization method can reveal the positions of the pipeline leakages, it can merely offer a poor resolution which is dozens of meters in size (i.e., the maximum −3 dB width is dozens of meters in size) [46,47]. For improving the localization resolution, a new TR localization method is developed in this paper. In this approach, the time reversed signals with specific coefficients are superposed. Since time reversed signals are multiplied by the coefficients, the localization functional value based on the proposed method decrease significantly with the movement of the observed point. Therefore, the resolution gets improved. For investigating the performance of the proposed method in passive detection application, a pipeline leakage localization experiment was conducted. For a 58 m pressurized pipeline, three manually controllable valves were utilized to simulate the leakages. Six piezoceramic transducers equipped on the pipeline, recorded the NWP signals generated by the three valves. The experimental results show that the proposed approach accurately reveals the leak locations with a maximum −3 dB width of 2 m. Meanwhile, the maximum −3 dB width of the conventional TR localization method is 20 m. That means the proposed approach can improve the resolution, compared to the conventional one.

## 2. Theoretical Approach of the Proposed Localization Method

**Figure 1:**
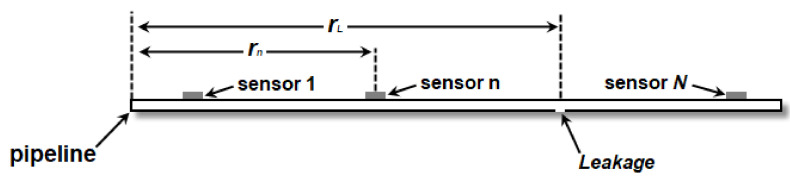
Model of the proposed method.

For corresponding to the pipeline leakage experiment, we describe the proposed method by using the model of pipeline. For a pressurized gas pipeline, a leakage will generate a negative pressure wave (NPW) which will propagate toward the two ends of a pipeline. The NPW can be detected by lead zirconate titanite sensors equipped on the pipeline wall. We assume that *N* transducers are used along a pipeline, and the $n^{th}$ transducer is located at $r_{n}$, as shown in Figure 1. We further assume that the leakage occurs at $r_{L}$. In this paper, plain symbols denote scalar quantities, whereas vectors and matrices are denoted by bold symbols.

For the purpose of convenience, we describe the proposed method in the time domain. Assume a NPW signal generated by the leakage is represented as x(t) and the leakage occurrence time is *t* = *T*. All transducers are synchronous. The leakage signal recorded by the $n^{th}$ transducer can be modeled as,

(1) $$y(t,r_{n},r_{L})=g_{r}(r_{n},r_{L},t)⊗x(t-T)$$

where “⊗” represents the convolution operation. The function $g_{r}(r_{n},r_{L},t)$ is the actual channel impulse response between the $n^{th}$ transducer and the leakage at $r_{L}$, obtained via measurement.

The time reverse signal $y(t,r_{n},r_{L})$, and the time-reversal version of Equation (1) can be represented as,

(2) $$y(-t,r_{n},r_{L})=g_{r}(r_{n},r_{L},-t)⊗x(-t-T)$$

A time reversal process is indeed equivalent to a correlation of impulse responses. Therefore, accounting for back-propagation from the $n^{th}$ transducer to the observation point $r_{k}$ in the monitoring domain, the time reversed signal at a point $r_{k}$ can be illustrated as,

(3) $$f(t,r_{n},r_{k})=g_{r}(r_{n},r_{L},-t)⊗x(-t-T)⊗g_{c}(r_{n},r_{k},t)$$

where $g_{c}(r_{n},r_{k},t)$ is the computational channel impulse response from the $n^{th}$ transducer to the point $r_{k}$.

Then, the so-called self-adaptive cancellation is used to process the time reversed signals: firstly, multiplying $f(t,r_{n},r_{k})$ by a coefficient ${\left(-1\right)}^{n}$. Secondly, summing over all time reversed signals multiplied by the coefficients. On the other hand, due to the various attenuation coefficients of the actual channels, the various sensors’ TR signals cannot cancel each other out via the superposition at the leak position. For eliminating that influence, the TR signals are normalized. The corresponding result can be written as,

(4) $$q(r_{k},t)=\sum_{n=1}^{N}{\left(-1\right)}^{n}f(t,r_{n},r_{k})/\max(f(t,r_{n},r_{k}))$$

Due to reciprocity principle, and assuming that the computational channel response function matches the measured data perfectly, namely $g_{c}(r_{n},r_{L},t)=g_{r}(r_{n},r_{L},t)$. Then, all time reversed signals $f(t,r_{n},r_{k})$ (n=1……N) will focus on the leakage location $\left(r_{k}=r_{s}\right)$ at the time t=−T, with similar waveform. Therefore, all time reversed signals multiplied by coefficients will cancel each other out at $r_{s}$ as the amplitude of $q(r_{s},t)$ approaches zero.

Since the time reversed signals cancel each other out at the leakage’s location, the signal energy in the leakage area is low than those in other areas. Therefore, the localization functional is designed as following,

(5) $$I_{p}(r_{k})={\left(\int \left|q(r_{k},t)\right|dt\right)}^{-1}$$

In the localization map, the localization functional value at leakage locations will be larger than those at other points.

**Figure 2:**
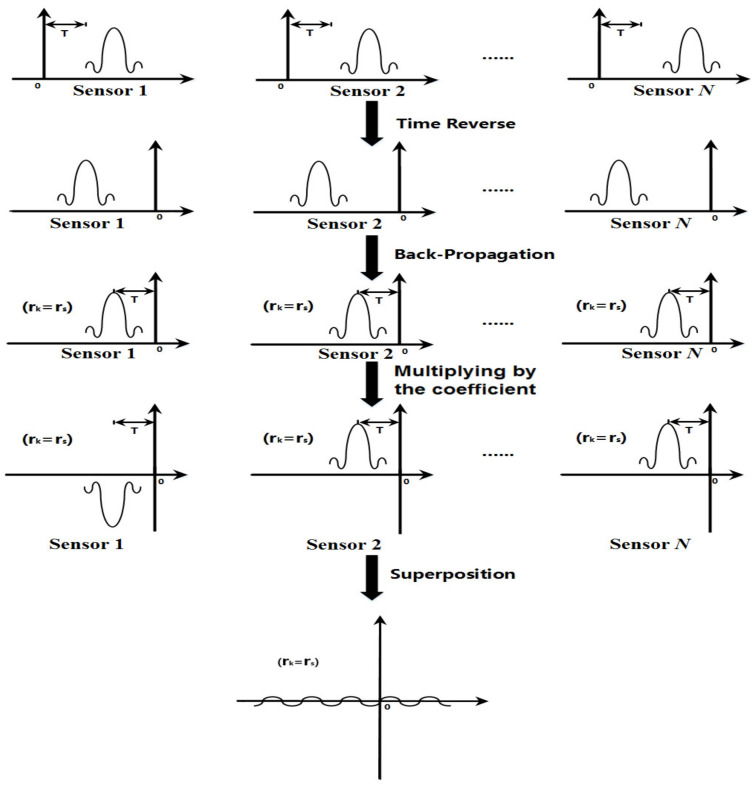
The diagram of signal processing based on the proposed method.

For the convenience of explanation, the diagram of signal processing based on the proposed method is shown in Figure 2. After time reversal and back-propagation, the time reversed signals focus on the leakage positions at the reference time *t* = −*T*. Due to the temporal focusing, those time reversed signals are the same to each other. Via multiplying the coefficient ${\left(-1\right)}^{n}$, part of the time reversed signals is upside down. Then, when the time reversed signals multiplied by coefficients are superposed, they cancel each other out. Therefore, via the self-adaptive cancellation, the amplitude of the final output signal at the leakage position approaches to minimum.

**Figure 3:**
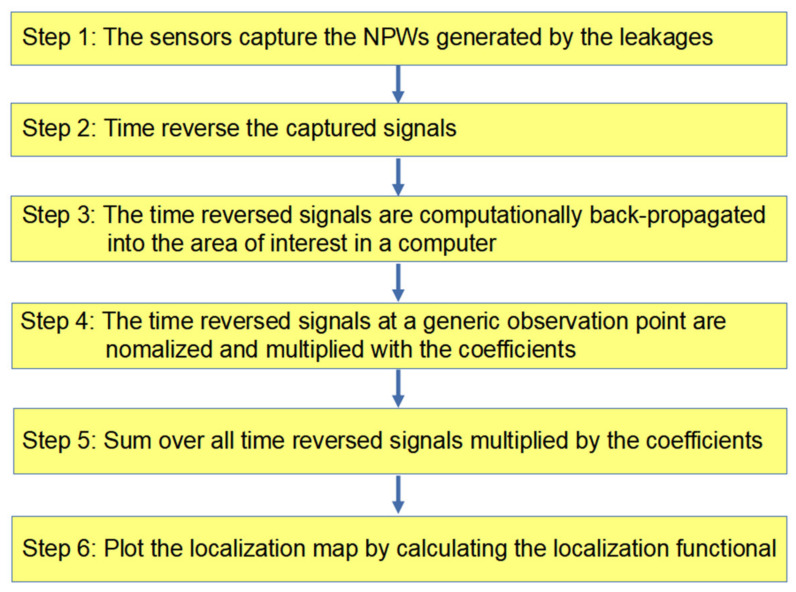
The procedure of the localization method.

Generally speaking, in the proposed method, the following steps are taken to localize the leakages, as shown in Figure 3. First of all, the sensors capture the NPW signals. Secondly, time reverse and back-propagate the captured signals (convolve the time reversed signal with channel impulse response in time domain). The time reversed signals at a generic observation point are normalized and multiplied with the coefficients (i.e., ${\left(-1\right)}^{n}$). Then, the sum of all time reversed signals is multiplied by the coefficients, as Equation (4) shows. Finally, plot the localization map is plotted by calculating the localization functional (i.e., Equation (5)).

## 3. Experiment

**Figure 4:**
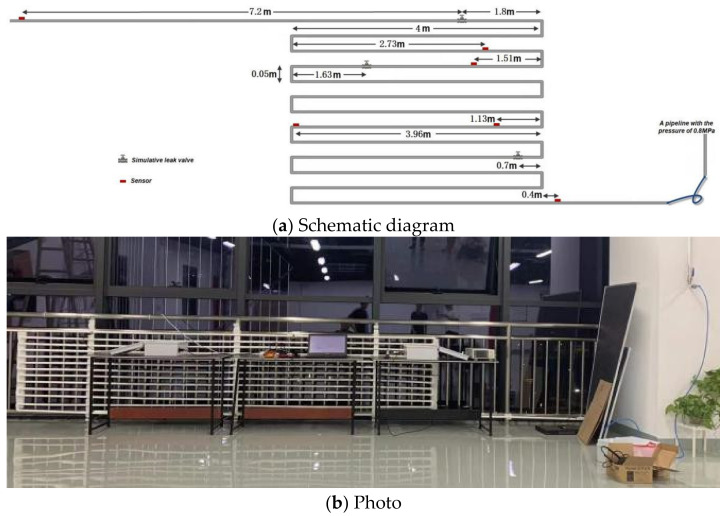
Schematic diagram and photo of the measured experiment.

As one kind of passive detection, a PVC pipeline leak experiment was executed. The pipeline was composed by a series of straight sections connected by twenty-four 90°-elbow connectors and twelve 0.05 m sections. The pipeline was fixed to a steel frame and connected to another pipeline with the pressure of 0.8 MPa, as shown in Figure 4. Six lead zirconate titanite (PZT) sensors whose size is 15 mm × 10 mm × 2 mm were directly mounted on the pipe wall and utilized to measure the local hoop strain change. Their special locations are listed at Table 1. The locations of three manually controllable valves equipped in the pipeline are shown in Table 2. The inner diameter of the valves is 12.7 mm. A leakage was simulated by opening any one of the valves. The data acquisition system is NI USB-6366 with the sampling rate of 2 MS/s. Since the distance between the first sensor and the last one was 58 m, the proposed method was utilized to monitor a pipeline of length = 58 m in this experiment. The host computer, which was utilized for localization computation, was equipped with i7-6700 CPU, 8 GB DDR3 memory.

The experiment was performed by taking the following steps: (1) The three valves were initially closed; (2) for each test, the pipeline was pressurized up to 0.8 MPa via the pipeline with the pressure of 0.8 MPa; (3) an event of leakage was created through opening one of the valves in the pipeline; (4) the data acquisition system recorded the NPW signals captured by PZT sensors; (5) by using the proposed method, the leakage position could be distinguished by analyzing the recorded signals.

Under the usual operating states of pressurized pipelines, the internal pressure of the pipeline is invariable and much higher than the external pressure [43]. When a leakage happens (i.e., a crack develops), the pipeline content escapes through the crack, thus, internal pressure drops significantly at the leakage point. The content in the pipeline shifts from both upstream and downstream towards the crack. Following the decrease of the internal pipeline pressure, the contraction of pipe wall decreases hoop strain variation on the surface of the pipe. The six piezoelectric sensors will detect the hoop strain variation.

## 4. Results and Discussion

**Figure 5:**
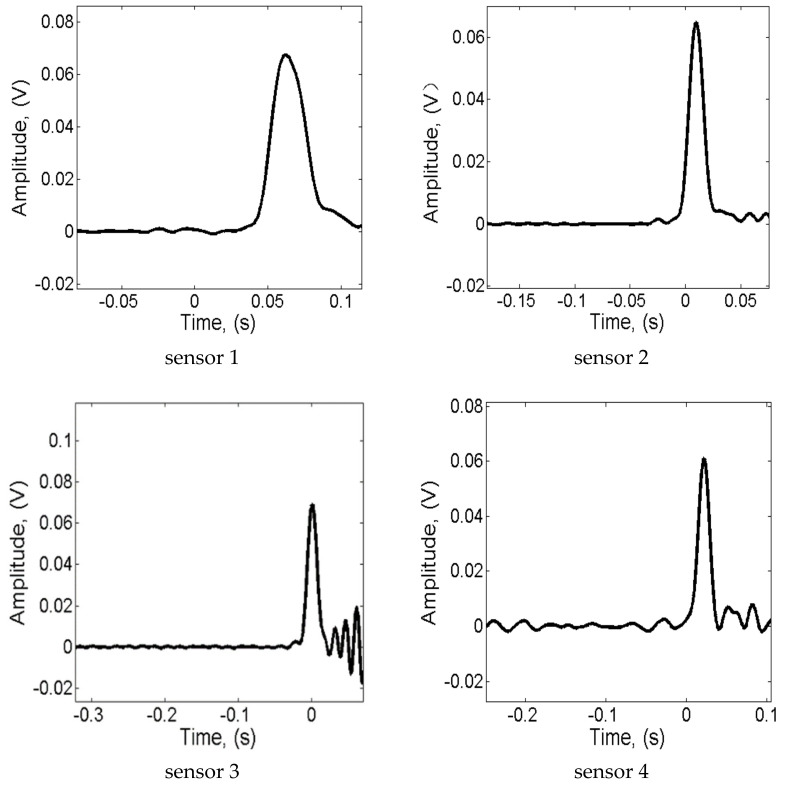

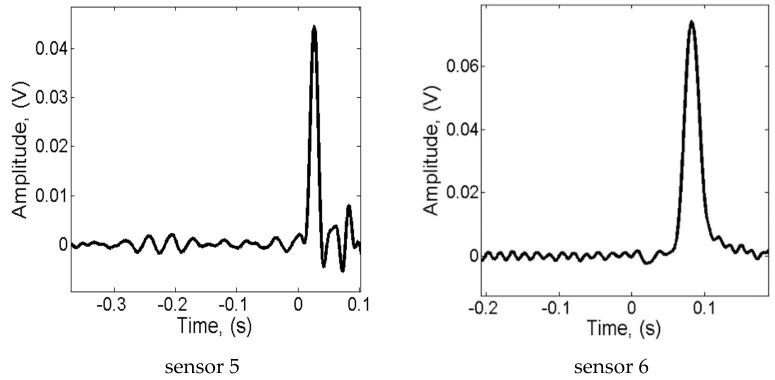
The NPW signals from leakage L2.

The typical waveform of the NPW captured by PZT sensors are shown in Figure 5. As aforementioned [43], for an undamaged pipeline, the amplitude is held at zeros as a result of the internal pressure keeping constant. Then, the pulse-like waveform is generated since the NPW reaches the sensors. The initial upward edge of the pulse indicates the drop of internal pressure associated with the NPW, while the downward edge means the internal pressure settling at a new baseline pressure. The peak of the pulse is the result of the NPW being arriving at the sensor’s position.

In the signal processing phase, all NPW signals generated the leakages are processed by using the conventional TR localization algorithms and the proposed algorithm, respectively. During re-constructing the leakage area, we made the channel impulse response $g_{c}(r_{n},r_{k},t)$ in Equation (3), by using the NPW propagation time of the corresponding channel. $g_{c}(r_{n},r_{k},t)$ can be written as the following [51]:

(6) $$g_{c}(r_{n},r_{k},t)=a_{n,k}\delta (t-\frac{distanc\;e_{n,k}}{v_{g}})$$

where δ(t) is the Dirac function, $distance_{n,k}$ is the distance between $r_{n}$ and $r_{k}$, $a_{n,k}$ is the attenuation coefficient of the computational channel from point $r_{n}$ and the localization point $r_{k}$. During the computational back-propagation, it can reduce computation cost for obtaining the attenuation coefficients and the waveform deterioration caused by the back-propagation to set the attenuation coefficients to 1 (i.e., $a_{n,k}$ = 1). Furthermore, as mentioned above, the normalization eliminates the influence of the attenuation coefficients of the channels (including the actual channels and the computational channels). Therefore, by using the parameter setting ($a_{n,k}$ = 1), the proposed method can obtain the results easily and accurately. The NPW velocity is considered as 300 m/s, which is also the theoretical NPW velocity reported in [42,43]. Furthermore, this value was chosen as it is situated in the upper margin of velocity estimation and for convenience of calculations [59,60].

Since the system recorded the NPW signals under voltage trigger mode, the leakage happened before the data acquisition system began to obtain the signals. Therefore, there is a time delay between the leakage occurrence time and the starting time of the received leakage signal, namely the parameter “*T*” of Section 2. After time reversing and re-transmitting the received NPW signals, the time reversed signals focus at the time *t* = −*T*. The conventional TR localization algorithm which used the signal values at the time *t* = 0 to re-construct the image of the leakage location is shown in following equation:

(7) $$I_{c}(r_{k})={\left.\sum_{n=1}^{N}f(t,r_{n},r_{k})\right|}_{t=0}$$

In order to focus the time reversed signals at the time *t* = 0, the time delay needed to be compensated. However, since the pipeline leakage occurrence time is unknown, the compensation cannot be done. In this case, the conventional TR localization based on the focal time will result in a wrong localization result.

Therefore, the conventional TR localization method based on the maximum of the time reversed signal is utilized to process the data for the purpose of comparison. The localization functional of the conventional TR localization algorithm based on the maximum value is represented as follows:

(8) $$I_{c}(r_{k})=\max\sum_{n=1}^{N}f(t,r_{n},r_{k})$$

**Figure 6:**
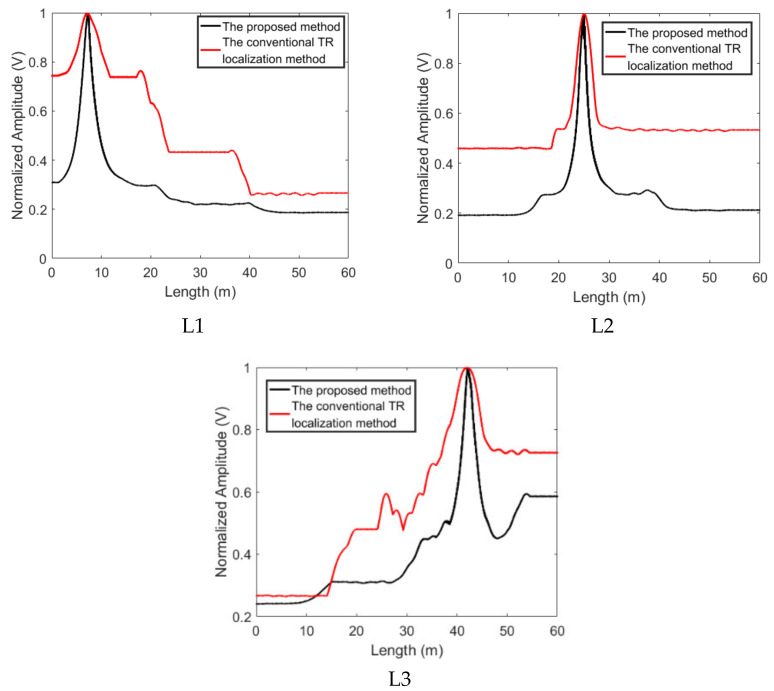
The localization results obtained by using the proposed method and the conventional TR localization method based on the maximum of the time reversed signal.

The results base on the proposed method and the conventional TR localization method are shown in Figure 6. The localization functional values are normalized, and the position of sensor 1 is considered as the starting point of the results. Since the distance between the first sensor and the last sensors is 58 m, the length of the pipe in a computer is set as 60 m for convenience, and the length of the grid is set as 0.2 m. As shown in Figure 6, the conventional TR localization method based on the peak of the time reversed signal can reveal the positions of the leakages. However, due to the long signal duration which is the result of the superposition of incident and reflected NPW, the time reversed signals still superposed with each other at the points beyond the leakage point. Therefore, the maximum value of the output signal attenuated slowly with the movement of the observed point, the localization functional values at a lot of points are very close to that at the leakage position. The leakage area revealed by the conventional TR localization method based on the maximum of the time reversed signal covers a large range. By using the conventional TR localization method, the −3 dB area of the L1 cover from length = 0 m to length = 19 m, the −3 dB area of the L2 is from length = 23 m to length = 27 m and the −3 dB area of the L3 ranges from length = 37 m to length = 60 m, as shown in Figure 6.

All time reversed signals will focus at the leakage positions with a similar waveform. That means that the time reversed signals can cancel each other out completely via Equation (4) at the leakage position. After integrating the superposed signals via Equation (5), the localization functional values at the leakage position will be larger than those at other positions. Due to the adoption of the temporal integration, we do not need to calculate the time delay which cannot be obtained. As shown in Figure 6, the plot based on the proposed method gives a good estimate of the leakage positions. By using the proposed method, the three estimated leakage positions are 7.4 m, 25 m, and 42.2 m, respectively. The maximum deviation is about 2.7% over a 58-m monitoring area. Furthermore, the resolution of the proposed method is superior to that of the conventional TR localization algorithm based on the peak of the TR signal. As shown in Figure 6, the lengths of the three leakages’ −3 dB areas are about 2 m, 2 m, and 4 m respectively. Obviously, the −3 dB area of the results based on the proposed method is much smaller than that of the conventional one [46].

**Figure 7:**
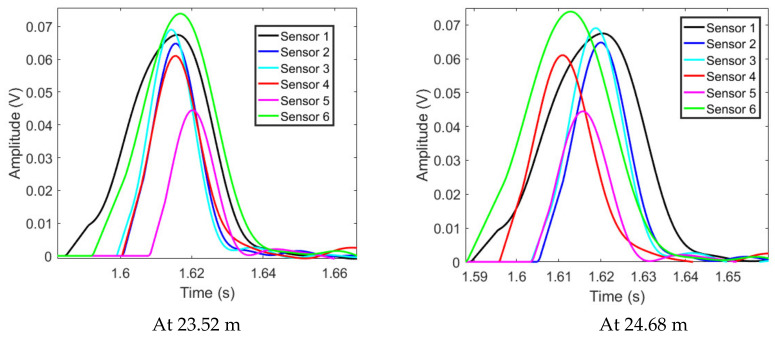

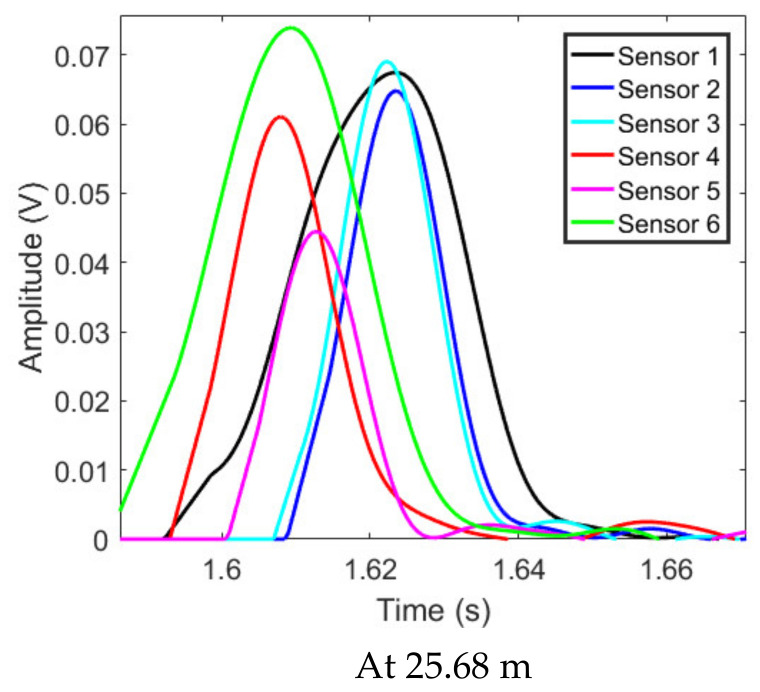
The L2’s time reversed signals.

**Figure 8:**
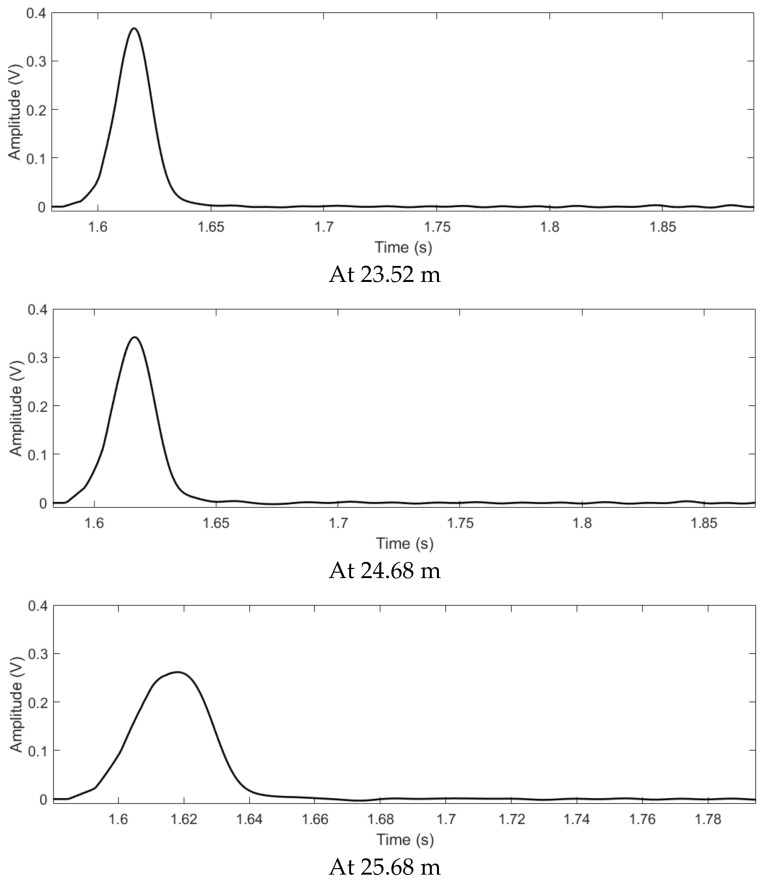
After the superposition, the L2’s signals of the TR localization method based on the maximum value.

**Figure 9:**
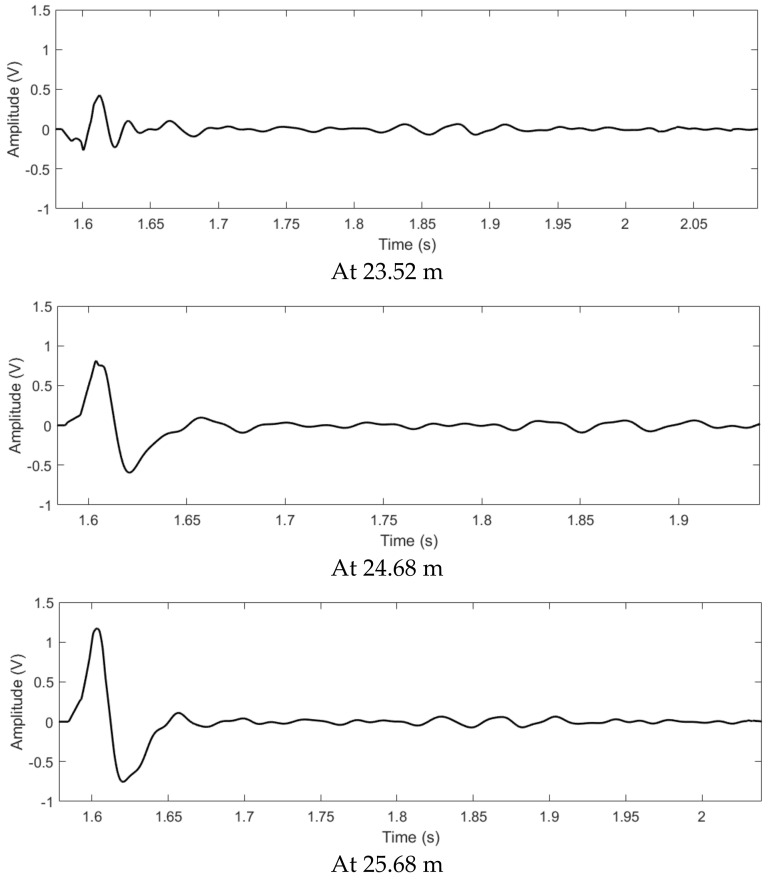
After the superposition, the L2’s signals of the proposed method.

At various observation points, the L2’s time reversed signals from the all sensors, the output signals of the proposed method and the output signals of the TR localization method based on the maximum signal are shown in Figure 7, Figure 8 and Figure 9. As shown in Figure 7, the time reversed signals completely overlap with each other at the leakage position. Furthermore, due to the long duration of the signals, most of the time reversed signals still superpose with each other at the points beyond the leakage. Therefore, the output signal of the TR localization method based on the maximum signal slowly attenuates at the points beyond the leakage position, as shown in Figure 8. On the other hand, by using the proposed method, most of the signals are canceled after the superposition. The output signal based on the proposed localization method rapidly enlarges at the points away from the leakage, as shown in Figure 9. The output signal of the proposed method at 24.68 m is twice larger than that at 23.52 m. However, the signal of the conventional TR localization method based on the maximum value only drops to 92%. At 25.68 m, the same phenomenon can also be found; the output signal of the proposed method at the leakage position is one third of that at 25.68 m, but the output signal of the conventional TR localization method based on the maximum value drops to 69%. Since the change of the output signal based on the proposed method is larger than that based on the conventional TR localization method, the −3 dB leakage area revealed by the proposed method contains less points, compared to that of the conventional TR localization method. Therefore, the resolution of the proposed method is better than that of the conventional TR localization method based on the maximum.

Generally speaking, by using the proposed method, the three estimated leakage positions are 7.4 m, 25 m, and 42.2 m respectively. That means that the maximum deviation is about 2.7% over a 58 m monitoring area. Furthermore, the lengths of the three leakages’ −3 dB areas are about 2 m, 2 m, and 4 m, which are superior to those of the conventional localization method. Therefore, the plot based on the proposed method gives a better estimate of the leakage positions, compared to the result based on the conventional one. That improvement is a result of the localization functional value based on the proposed method decreasing significantly with the movement of the observed point. Besides, the estimated localization errors of the proposed method are the result of the NPW reflecting off the ends of the pipeline. The superposition of incident and reflected NPW postpones the peak of the NPW signal. Consequently, the estimated leak locations shift from the actual locations slightly. However, the estimated localization error of 2.7% is less and comparative.

## 5. Conclusions

When the conventional TR localization methods are employed for pipeline leakage localization, they suffer from some issues, such as a low resolution. In this paper, a novel time reversal localization method is designed for localizing pipeline leakages. Via the self-adaptive cancellation and the temporal integration, the proposed localization method can reveal NPW sources with an accurate and high-resolution result. We applied the proposed method for leakage localization in a pressurized pipeline with PZT sensors. The results indicate the proposed method can localize the leakages with a high accuracy. Furthermore, the resolution of the proposed approach can be ten times that based on the conventional TR localization method.

## Figures and Tables

**Table 1:** Specific positions of the sensors.

Sensors	Distance from Sensor 1 (Unit: m)
Sensor 2	15.83
Sensor 3	18.66
Sensor 4	34.48
Sensor 5	37.31
Sensor 6	58

**Table 2:** Specific locations of the leakages.

Leakages	Distance from Sensor 1 (Unit: m)
Leakage 1 (L1)	7.2
Leakage 2 (L2)	23.52
Leakage 3 (L3)	42.15

## Data Availability

Not applicable.
